# Feasibility of Health Literacy Tools for Older Patients in the Emergency Department

**DOI:** 10.5811/westjem.2020.5.46594

**Published:** 2020-08-20

**Authors:** Matthew J. McGuinness, Joshua Bucher, James Karz, Carla Pardee, Laryssa Patti, Pamela Ohman-Strickland, Jonathan V. McCoy

**Affiliations:** * Rutgers Robert Wood Johnson Medical School, New Brunswick, New Jersey; † Rutgers Robert Wood Johnson Medical School, Department of Emergency Medicine, New Brunswick, New Jersey; ‡ Rutgers School of Public Health, Piscataway, New Jersey

## Abstract

**Introduction:**

This study evaluates the feasibility of using a volunteer research associate (RA) to administer two separate health literacy assessment tools in the emergency department (ED), specifically in an older population of patients. The outcomes measured were administration time and interruptions.

**Methods:**

Using a prospective, cross-sectional study with a convenience sample, adult patients over the age of 55 presenting between June–August 2018 to one urban, academic ED were evaluated by a volunteer RA using either the Newest Vital Sign (NVS) or the Short Assessment of Health Literacy (SAHL). All patients 55 years of age or older who consented to participate were included. We excluded from this study the following: patients with dementia or other disability involving reading, speech, or cognitive function, as noted in their medical record or by their attending physician; prisoners; and those subjectively deemed in extremis or too ill to participate by their attending physician.

**Results:**

Health literacy was assessed in 202 patients using either the NVS or SAHL. Mean time of administration was 214.0 seconds for the NVS, and 206.8 for the SAHL. The maximum time of administration for the NVS was 563 seconds, compared to 607 seconds for the SAHL. We found that 95.2% of NVS and 93.9% of SAHL tests incurred no interruptions during administration.

**Conclusion:**

No significant difference was found between the length of time needed to administer the NVS or SAHL to older patients in the ED. Both tools averaged an administration time of around three to four minutes, and neither incurred regular interruptions to its administration by a volunteer RA. Further study is needed to assess validity of these tools in an ED setting.

## INTRODUCTION

In their extensive 2004 report, the Institute of Medicine defined health literacy as “the degree to which individuals can *obtain*, *process*, and *understand* the basic health information and services they need to make appropriate health decisions.”^1^ As healthcare systems change, patients frequently remain responsible for adhering to treatment protocols and seeking proper follow-up. Adequate health literacy is key to achieving proper compliance to medication use and has been shown to improve healthcare outcomes.^2^,^3^ Health literacy is complex, and often described as consisting of a variety of components, including literacy and numeracy. The term “literacy” is used to explain aspects of language involving reading, writing, speaking, or listening, while “numeracy” refers to quantitative aspects of health literacy, including basic mathematical operations.^1^ Issues of health literacy may be further complicated by language barriers or other barriers to communication. For these reasons the American Medical Association has encouraged continued research in health literacy.^4^,^5^

Among the variety of healthcare settings, the emergency department (ED) is a focal point of health literacy research. The 2003 National Assessment of Adult Literacy estimated that around 36% of adults had basic or below basic health literacy levels.^4^ A review of ED patients showed that low health literacy may be present in up to 40% of patients.^3^ Care in the ED involves rapid decision-making and swift communication between providers and their patients. Low health literacy in the ED has been associated with worse healthcare outcomes and increased recidivism.^3^,^6^

One challenge of assessing health literacy in the ED is doing so effectively and efficiently. The ideal health literacy assessment tool for an ED is one that is easily understood by both the patient and those who may administer it (including volunteers, technicians, nurses, physician assistants, and physicians), takes little time to administer, is well studied, validated, and considers various demographic factors such as age or language spoken. Various tools have been developed, but many are primarily for or only available to English-speaking patients.^7^,^8^ Common assessment tools used for both English and Spanish speakers include the Newest Vital Sign (NVS) and Short Assessment of Health Literacy (SAHL).^6^, ^9^,^10^ Due to their relative ease of use, short time to administer, and availability in both English and Spanish, these tools are good prototypes for use in the ED and should be further evaluated. While these tools have been found to be effective in their ability to assess health literacy, concern has been raised about their efficiency, as well as their utility in measuring health literacy among the elderly.^11^ Therefore, further investigation is needed to determine the feasibility of administering the NVS and SAHL in this particular segment of the population in the ED.

The purpose of this study was to determine the extent to which these two health literacy assessment tools (the NVS and SAHL) can feasibly be performed by a volunteer research associate (RA) to assess health literacy of older ED patients. This study included both English and Spanish speakers to analyze the performance of these tools in a diverse patient population.

## METHODS

### Study Design and Population

This was a prospective, convenience sampling, cross-sectional study. The 2017 American Community Survey estimates 49.3% of the population of the study location speaks Spanish.^12^ Verbal consent was obtained from all patients. This study was conducted at an urban, academic adult ED with approximately 80,000 total annual visits. Patient recruitment occurred between 9 am and 5 pm, primarily on weekdays based on availability of a medical student acting as a volunteer RA. No direct recruitment of volunteer RAs occurred during this study. In preparation for this study, the RA had extensively reviewed and helped prepare the administration tools, a process that took no more than three hours, and practiced with colleagues before administering to patients.

**Population Health Research Capsule**

What do we already know about this issue?

Low health literacy has been associated with worse health outcomes and increased recidivism in the emergency department (ED). Up to 40% of ED patients may have low health literacy.

What was the research question?

Can a volunteer research associate (RA) feasibly administer health literacy assessments to older patients in the ED?

What was the major finding of the study?

Administration of a health literacy tool by a volunteer associate takes 3–4 minutes, and is not regularly interrupted.

How does this improve population health?

Regular assessment of patient health literacy in the ED is feasible, and may be best achieved with the help of volunteer RAs.

Patients enrolled were identified by the RA as being 55 years of age or older by electronic health record review and spoke English or Spanish. Exclusion criteria were as follows: patients 90 years of age or older; any patient deemed to be under significant distress by their attending physician; prisoners; and patients who had an altered mental status for any reason. Patients were also excluded if their primary language was not English or Spanish. Patients 90 years of age and older were excluded in order to maintain institutional review board (IRB) compliance and avoid collection of protected health information along given the demographic data being collected. IRB approval was obtained from the IRB board of Rutgers University.

### Protocol

Upon identifying a patient eligible for participation in this study, the RA approached the care provider most closely associated with the patient (either a physician assistant or physician) to ask about enrolling the patient. The care provider identified whether the patient met any exclusion criteria (critical illness, physical distress, or alteration of mental status, dementia, or other disability involving reading, speech, or cognitive function). If the patient was deemed eligible by the physician assistant (PA) or physician caring for the patient, the RA then administered the assessment during the patient’s ED visit. Data collection did not interfere with patient care. All staff in the ED were made aware of this study and encouraged to interrupt assessments or research activity if patient care was required. Results of the health literacy assessment were not reported to any physician and PA caring for the patient. Despite the limited hours of potential enrollment, it is believed that nearly every patient meeting potential inclusion criterion was identified and screened during these times, although we collected no data explicitly examining percentage of eligible patients recruited.

Each encounter with an enrolled patient consisted of a survey of demographic information and an assessment of their health literacy using one of the standardized aforementioned tools (either the SAHL or NVS). A computerized, random-number generator, with 1 representing the NVS and 2 representing the SAHL, was used to randomly assign which tool would be administered to each patient. The survey was conducted in the patient’s preferred language. To standardize the experience for each patient, the RA attempted to minimize questions from patients about the test; however, this may have led to concerns in patient’s understandings of the test material. To assess the efficiency of each survey, we recorded the time elapsed to administer them, and the frequency of interruptions. Family or friends visiting the patient were advised not to assist the patient during the survey and health literacy test.

### Screening Tools and Outcomes

The health literacy screening tools used included the SAHL^10^ and NVS,^11^ both of which were previously validated by comparison to the Test of Functional Health Literacy in Adults (TOFHLA), the most frequently used tool across outpatient settings. The TOFHLA is widely regarded as one of the most validated of all health literacy assessments. Both initial validation studies for these tools took place in outpatient, primary care settings.^10^,^11^

### Data Analysis

Distributions of age, number of seconds of interruptions, and time taken were investigated using histograms and means. Frequencies were reported for the distribution of categorical variables. We compared significance in time of administration differences between scoring groups using t-tests.

## RESULTS

Of 202 patients enrolled in this study, 104 patients were randomly assigned to and administered the NVS while 98 were randomly assigned to and administered the SAHL. Table 1 demonstrates demographic data of each study group. The mean age of patients who took the NVS was 68.1 years. The mean age of patients who took the SAHL was 69.2 years. Spanish speakers represented 19.2% of those administered the NVS, and 16.3% of those administered the SAHL.

Table 2 includes all data associated with time of administration and interruptions to administration. The NVS averaged a mean time of administration of 214.0 seconds (3.57 minutes), while the SAHL averaged a mean time of 206.8 seconds (3.45 minutes). There was no significant difference in time of administration between the NVS and SAHL (t = 0.6379, p = 0.5242). The longest time needed to administer the NVS was 563 seconds (9.38 minutes), compared to 607 seconds (10.1 minutes) for the SAHL; 95.2% of all NVS tests and 93.9% of all SAHL tests incurred no interruptions during administration. For both the NVS and SAHL, interruptions lasted a mean of approximately five seconds (5.54 for NVS, and 4.96 for SAHL).

## DISCUSSION

Concern continues to grow that health literacy issues may be an epidemic affecting health outcomes and healthcare costs.^3^,^13^–^16^ Low health literacy can impact a variety of issues in healthcare including patient decision-making and understanding discharge instructions.^17^ The best way to address these issues remains a serious discussion for healthcare providers and administrators, and a consistent means of measuring and analyzing patient health literacy is needed. Research interests regarding the use of health literacy tools in the ED has grown, but these studies may not adequately represent older adults, a population of obvious importance in the acute care setting.^7^,^11^ Ideally, a tool for evaluating the health literacy of older adults in the ED should be simple, efficient, and accessible to a diverse patient population. Having an awareness of a patient’s health literacy, or lack thereof, allows physicians, PAs, nurses, and all care providers to identify those who may need additional support with regard to decision-making and follow-up care.

Few studies have evaluated the feasibility or efficiency of implementing health literacy tools within the ED. Carpenter et al examined feasibility of health literacy tools in the ED, but focused on the NVS, Rapid Assessment of Adult Literacy in Medicine (REALM), and the short version of TOFHLA tests among average-aged, English-speaking patients.^8^ Our study focused primarily on the health literacy of older patients, including both English and Spanish speakers, as well as use of a volunteer associate in administration of health literacy tools. Other studies of health literacy assessments in the ED have not exclusively featured an older population, and many have not used both English- and Spanish-speaking patient populations.^8^,^11^,^13^,^18^ In addition, our sample population represented diversity in level of education, which may be a more accurate depiction of ED patients.

Given the complex, busy environment of ED care, health literacy tools are unlikely to be part of routine assessment by physicians, PAs, and other providers. Therefore, efficient, reliable tools may need to be performed by other support staff, with ED volunteers representing a potentially useful group to provide this role. This study demonstrates that use of a volunteer in the ED setting could allow for rapid assessment of health literacy of older patients. This information could prove useful for physicians and ED staff in their decision-making and communication with patients given health literacy’s impact on patient outcomes and recidivism.^3^,^6^,^17^

Both the SAHL and NVS are common tools for assessment of health literacy and show similar ease of use with regard to time of administration for older patients. Neither test was lengthy enough to incur significant interruptions, an important consideration concerning the environment of an ED. A lack of interruptions, despite encouragement of ED staff to interrupt testing for patient care, implies that administration of these tools by volunteer staff is unlikely to impede patient care. Although not actively measured, the physicians and PA involved with this study noted no impact in their ability to provide care because of these assessments. Both tests averaged a time between three and four minutes to administer, an implication that both have similar efficiency in an ED setting. Times of greater length could make either test inappropriate in the care of patients in the ED setting, as it would discourage routine assessment of health literacy, even by a volunteer or staff member not directly providing care. Of note, the longest administration time for both tests was approximately 10 minutes in length. In many EDs, 10 minutes is likely to be too lengthy for a health literacy assessment. This could reflect the need for a cutoff point among health literacy assessments in the ED – an area for future study or consideration in development of new tools.

Defining the most efficient and clinically useful health literacy assessment tool remains an issue of importance to improving patient care.^7^ This study emphasizes the feasibility of these two assessments, the NVS and SAHL, in the ED setting, as well as administration of these tools by a volunteer RA. However, ease of use is only one important criterion for an appropriate health literacy tool. Future study in this area will need to emphasize validity of these tools in this setting, and among specific patient populations such as the elderly or Spanish-speaking patients. Further development may be needed to generate tools that will maximize efficiency for this setting, while retaining high sensitivity and specificity for adequate health literacy. Finally, the success of a volunteer administering tests in this limited setting presents a possibility for similar roles for medical students and volunteers in the ED setting. Additional development of programs such as this could involve these volunteers to help gather important information on social determinants of health to better patient care.

## LIMITATIONS

Despite random assignments, selection bias was possible given our exclusion criteria. This study focused on older patients at a single center and excluded several groups frequently seen in the ED population. The most apparent of these are individuals who were deemed too critically ill to be interviewed. The determination of when a patient was “critically ill” was made by a patient’s physician or PA on a case-by-case basis, introducing the possibility that certain patient populations were improperly excluded. In addition, the study excluded individuals with dementia or other neurologic disability. If more individuals with unknown or unreported cognitive decline or dementia were more prevalent in receiving a certain tool, it could introduce bias that interferes with the scores or time taken to administer that tool. Sampling occurred via convenience sampling. While this carries some risk of selection bias, it is the most efficient method for studies based in the ED.

Enrollment was restricted to a limited period based on the availability of a volunteer, and did not include all hours in which patients meeting inclusion criteria would present to the ED. The percentage of patients meeting criteria who were evaluated for potential enrollment was not assessed. No direct measures were used by research staff to determine ongoing testing quality apart from initial training. Nor were any ED staff objectively surveyed regarding their views about how administration of tests in this study impacted care in the ED. While discouraged from contributing, visitors with the patient could have influenced patient responses. In addition, only a single RA was available to administer the health literacy tools, increasing the possibility to bias administration of the tools. This RA speaks Spanish, but was not certified as a medical interpreter in Spanish at the time of data collection, which may raise concern about patient understanding of instructions and tools. However, instructions given in Spanish are directly provided by the NVS and SAHL tools for the possibility of use by non-fluent personnel, and overall instructions about this study were written by a certified medical interpreter.

## CONCLUSION

This study examined feasibility of the NVS and SAHL as tools to examine health literacy of an older population of ED patients. This study successfully employed a volunteer research associate to administer these health literacy tests in an ED setting. No major differences were seen in the amount of time needed to administer these tools by a volunteer RA across the entire study population. In addition, there were instances in which both tools exceeded nine minutes to administer. This encourages continued study into finding more efficient tools in evaluating health literacy, especially for older patients. Further study of these tools, and programs to implement their use in the ED, must highlight their validity and overall effectiveness in assessing health literacy in an ED setting.

## Figures and Tables

**Table 1:** Demographic data of patients administered survey tools to assess health literacy.

Demographic	NVS	SAHL
Total number administered	104	98
Mean age of patients	68.1	69.2
Spanish forms administered (%)	27 (26%)	24 (24.5%)
Number of female patients (%)	53 (51%)	51 (52%)
Number with 4-year degree or higher education (%)	24 (23.1%)	33 (33.7%)

*NVS,* newest vital sign; *SAHL,* short assessment of health literacy.

**Table 2:** Summary statistics for seconds to complete and time of interruptions for NVS and SAHL health literacy tools.

Time	NVS	SAHL	
Mean TOA	214.0 seconds	206.8 seconds	p = 0.5242
SD of TOA	75.97	84.4	
Min TOA	106.0 seconds	106.0 seconds	
Max TOA	563.0 seconds	607.0	
CI for mean	199.2–288.8 seconds	189.9–223.7 seconds	
Percentage of tests with no interruption	95.2%	93.9%	
Mean time of interruptions per administration	5.54 seconds	4.96 seconds	

*NVS,* newest vital sign; *SAHL,* short assessment of health literacy; *TOA,* time of administration; *SD*, standard deviation; *CI*, confidence interval.
